# Status of Selenium and Other Essential and Toxic Elements in Oregon Grazing Sheep

**DOI:** 10.3390/ani15121799

**Published:** 2025-06-18

**Authors:** Daniella Hasan, Christopher J. Russo, Katherine R. McLaughlin, Gene Pirelli, Massimo Bionaz

**Affiliations:** 1 Department of Animal and Rangeland Sciences, Oregon State University, Corvallis, OR 97331, USA; 2 College of Earth, Ocean and Atmospheric Sciences, Oregon State University, Corvallis, OR 97331, USA; 3 Department of Statistics, Oregon State University, Corvallis, OR 97331, USA

**Keywords:** selenium, sheep, deficiency, trace minerals, cobalt, copper, normative values

## Abstract

Mineral imbalances in sheep can have significant health and economic consequences, yet regional assessments of trace mineral and toxic element status are limited. Oregon is an endemically selenium (Se)-deficient region, but a statewide evaluation has not been conducted. This study assessed whole blood concentrations of 18 elements in 370 clinically normal ewes from 56 farms across Oregon to determine the prevalence of deficiencies and potential toxic exposures. A deficiency threshold of <120 ng Se/mL whole blood was used. We found that 28% of animals were Se-deficient, and 27% of farms had an average whole blood Se below this threshold. No animals reportedly showed overt clinical signs of deficiency or toxicity, and no excessive exposure to toxic elements such as arsenic, lead, or cadmium was found. We also compared mineral concentrations in blood collected using standard EDTA tubes versus mineral-free EDTA tubes, and in whole blood versus plasma, to assess alternative sampling strategies. Strong correlations (r > 0.9) for Se, cobalt (Co), and molybdenum (Mo) between plasma and whole blood allowed for the derivation of conversion equations. These findings provide updated insights into the mineral status of Oregon sheep, contribute to whole blood reference data, and support practical alternatives to improve trace mineral testing in field conditions.

## Simple Summary

This study evaluated blood mineral levels in 370 healthy sheep from 56 farms across Oregon, a region known for selenium (Se)-deficient soils. The goal was to assess the prevalence of mineral imbalances and identify practical sampling methods for on-farm monitoring. We found that 28% of sheep were Se-deficient, and 27% of farms had average Se levels below the deficiency threshold (<120 ng/mL whole blood), despite the absence of clinical symptoms. No farms showed toxic levels of arsenic, lead, mercury, or cadmium. Blood collection in standard purple-top EDTA tubes produced results equivalent to mineral-free tubes, offering a more accessible option for producers. Additionally, whole blood may be suitable for measuring Se, cobalt, and molybdenum when plasma separation is not feasible. Our study analyzing the whole blood concentration of 18 different minerals contributes valuable data toward establishing whole blood reference ranges for sheep. These findings improve our understanding of sheep mineral status in the region and highlight simple, cost-effective approaches to enhance routine mineral surveillance, allowing for earlier intervention and improved flock health.

## 1. Introduction

An evaluation of sheep trace mineral and heavy metal status has not previously been performed in Oregon despite unique challenges with geographically associated mineral deficiency. Selenium (Se) is an essential micromineral that plays a crucial role in various physiological processes in mammals, including antioxidant defense, thyroid hormone metabolism, and immune function, and Oregon forages are known to be deficient in Se due to low soil content [1,2,3,4,5]. Low levels of dietary Se are known to cause deficiency of Se in sheep, which may lead to severe consequences, such as white muscle disease, a degenerative myopathy typically affecting lambs that is characterized by progressive weakness and high mortality rates of up to 50% [6,7]. Oregon has been recognized as an endemically Se-deficient region since the 1960s [7,8]. Early assessments of Se availability primarily relied on soil and forage Se concentrations [9,10]; however modern livestock systems have introduced complexities that make such generalizations less straightforward. Sheep producers in Oregon have implemented various strategies to mitigate Se deficiency, including the use of Se-fortified forages, imported feedstuffs, fortified concentrates, dietary mineral supplements, and routine Se injections.

While the importance of Se has been reinforced by the well-documented health issues incurred by Se-deficient humans and animals, other microminerals also play essential roles in livestock health and productivity [10,11,12,13,14,15,16]. Deficiencies in microminerals such as copper, cobalt, and manganese may also impair sheep health and productivity [17]. However, the status of these microminerals in sheep populations across Oregon remains largely unexplored. While Se is undoubtedly a vital nutrient, it should not be overlooked that other trace minerals and heavy metals play roles in sheep health and production. Moreover, the bioaccumulation of minerals—both toxic and essential—has implications for human health when livestock enter the food supply chain [18]. Exposure to toxic elements (e.g., lead, cadmium, arsenic) in sheep may present a hazard to human health depending on the extent of environmental contamination, dietary intake, and bioavailability of these elements in animal tissues. Despite the known implications of mineral accumulation in livestock for both animal and human health, the routine testing of mineral levels in animal products (especially in the “farm to table” market) is not a standard regulatory requirement in the U.S. This lack of oversight potentially facilitates a route of dietary exposure to both essential and toxic elements in lamb consumers. Thus, monitoring mineral status can be used to evaluate the nutritional quality and safety of animal products.

The consequences of micromineral deficiency in sheep can be broad, impacting not only animal health and production but also the economic viability of sheep farms. Inadequate mineral supplementation can lead to suboptimal growth rates, reduced wool quality, and increased susceptibility to diseases, ultimately resulting in financial losses for producers [19]. Previous studies have highlighted the importance of mineral supplementation in sheep under controlled settings [14,19,20,21], which may not fully reflect industry practices or account for diverse factors such as grazing region and supplementation strategies.

Screening for deficiency can be challenging as animals may not exhibit symptoms of deficiency until they are in dire straits. There are limitations with obtaining liver or plasma samples, and there is a lack of available clinical reference values for whole blood despite it being a very practical matrix for producers. Liver testing is more invasive than blood testing and requires skilled techniques to avoid complications.

One of the barriers to accessing mineral deficiency screening in sheep is obtaining plasma samples. Extracting plasma from whole blood requires the ability to centrifuge blood on-site or with local access to veterinary services. Establishing whole blood reference ranges for minerals commonly measured in serum or plasma may alleviate this burden. Most reference values are based on serum, but for most minerals, plasma and serum values are assumed to be the same [22,23,24]. Resolving the need to establish whole blood trace mineral reference ranges would make deficiency screening much more accessible and cost-effective for producers. With this purpose, we conducted a comparison between whole blood and plasma values of several microminerals through a veterinary diagnostic lab to derive predictive whole blood reference values from pre-established plasma reference values. Another obstacle for producers is obtaining certified mineral-free EDTA K2 blood tubes, which are considered the best practice for blood mineral testing to avoid contaminations that may skew the results. Standard purple-top EDTA K2 blood tubes are more readily available and affordable. We also compared the mineral concentrations of blood collected from animals in standard EDTA K2 tubes and trace-mineral-free EDTA K2 tubes.

The primary objective of this study was to assess the mineral status of sheep grazing in Oregon, identifying the prevalence of deficiencies and potential toxic exposures. The secondary objective was to improve access to trace element testing for producers by determining the feasibility of using standard EDTA tubes instead of trace element-free tubes and whole blood instead of plasma.

## 2. Materials and Methods

### 2.1. Study Design and Animal Use

Experimental procedures used in this study were approved by the Institutional Animal Care and Use Committees (IACUC) of Oregon State University (protocol #2022-0295).

Participating volunteers were recruited via regional social media groups, word of mouth, email, and promotion by newsletter to members of the Oregon Sheep Growers Association and the American Sheep Industry Association. The inclusion criteria for farms were as follows:

- Have at least 10 ewes on-site.
- Have been feeding a local pasture diet with or without supplementation for the past three consecutive months, and the diet must omit any grain or forages grown outside of a 10-mile radius.
- Be able to provide a mineral tag if they are feeding a mineral supplement.

Once the farm qualified to participate, the selection of ewes was made with the following criteria:

- Open and not lactating.
- Not have received an injection of Se in the past three months (e.g., BO-SE, MU-SE).
- Have lambed once before and are not known to be currently experiencing infertility.
- Healthy and not recently been ill.
- Between two and seven years of age.
- Body condition score between 2 and 5 on a five-point scale.

### 2.2. Definition of Terms

Terms were adapted from Mineral Levels in Animal Health (Puls, 1988) [25].
Deficient: Levels at which clinical or pathological signs should be apparent as described in the literature.
Normal: Used where deficiencies are unknown; indicates normal background levels.
Adequate: Levels sufficient for optimum functioning of all body mechanisms with a small margin of reserve to counteract commonly antagonistic conditions.
Toxic: Levels at which subclinical, clinical, or pathological signs of toxicity would be expected to occur.

### 2.3. Blood Collection

We evaluated the whole blood concentrations of 18 minerals in 370 well-appearing domestic ewes grazing on 56 Oregon farms to gauge the prevalence of nutritional deficiency and identify potential toxicities. These minerals included aluminum (Al), arsenic (As), boron (B), barium (Ba), beryllium (Be), cadmium (Cd), cobalt (Co), chromium (Cr), copper (Cu), iron (Fe), mercury (Hg), manganese (Mn), molybdenum (Mo), lead (Pb), selenium (Se), thallium (Tl), vanadium (V), and zinc (Zn). Additionally, we compared mineral concentrations in blood samples collected using standard EDTA tubes versus mineral-free EDTA tubes and using whole blood instead of plasma.

Blood was collected via jugular venipuncture using a 20-gauge hypodermic needle and trace mineral-free royal blue top EDTA K2 tubes (CAT# 368381; BD Vacutainer; Franklin Lakes, NJ, USA) between August 2023 and January 2024. For 12 sheep in 4 farms, following collection by a researcher into the blue-top tube, blood was collected into purple-top EDTA K2 tubes (CAT# 366643; BD Vacutainer; Franklin Lakes, NJ, USA). Either blood was collected by a member of the research team, or blood collection supplies were mailed to the farm with instructions on how to perform the blood draw, along with a shipping label to send the collected blood to our laboratory at room temperature.

For a subset of samples (farm #6, #11, #31, #43), whole blood and plasma were collected by a researcher for cross-comparison of our results with the Texas A&M Veterinary Diagnostic Lab and, using the same laboratory, to compare results from plasma and whole blood from the same animals. Samples were shipped in aliquots at ambient temperatures in February 2024, following laboratory instructions. Given the time of year, exposure to excessive temperatures during transit was not anticipated. A portion of the blood and plasma was reserved to submit to the Texas A&M University Veterinary Diagnostic Laboratory for total Co, Cu, Fe, Mn, Mo, Se, and Zn concentration.

### 2.4. Whole Blood and Plasma Digests

To collect plasma, blood was centrifuged at 2000× *g* for 10 min at room temperature within four hours of blood collection, transferred via disposable transfer pipette to 1.7 mL Eppendorf tubes, and frozen at −20 °C. Upon arrival at the lab, whole blood was frozen at −20 °C in royal-blue collection tubes until digestion.

For digestion, 500 μL of whole blood or plasma was transferred to a 15 mL polypropylene conical centrifuge tube (CAT# 339650; ThermoScientific; San Jose, CA, USA). Then, 100 μL of indium (CAT# MSIN-10PPM; Inorganic Ventures; Christiansburg, VA, USA) 100 ng/mL was added to each tube. This was followed by adding 1125 μL of home-distilled concentrated nitric acid and 1125 μL of lab-grade 30% hydrogen peroxide (CAT# HX0635-3; Millipore Sigma; Burlington, MA, USA). Tubes were tightly capped and allowed to pre-digest at room temperature for one hour. Tubes were then placed in a 90 °C bead bath and allowed to digest until a clear digest was achieved, approximately 6–8 h. Afterward, tubes were allowed to cool to room temperature, and samples were diluted with 10 mL Milli-Q distilled water.

### 2.5. ICP/MS Analytical Method and Quality Assurance

Trace element concentrations in whole blood and serum samples were carried out at the WM Keck Collaboratory for Plasma Spectrometry at Oregon State University. The analyses were conducted on a ThermoScientific iCAP-RQ quadrupole ICP-MS (CAT# BRE731416; ThermoScientific; San Jose, CA, USA) configured with a robust skimmer cone insert and operated in standard (STDR) and kinetic energy discrimination (KEDR) modes. In this study, Be9, B11, As75, Mo98, Cd111, Ba137, Hg200, Tl205, and Pb208 were analyzed in STDR mode, and Al27, V51, Cr52, Mn55, Fe57, Co59, Cu65, Zn66 and Se77 were analyzed in KEDR mode, with the two modes of operation occurring in sequence. Analyses were programmed such that KEDR measurements occurred first, followed by STDR measurements. Operating in this order allows the collision cell to stabilize for the entirety of the 45 s sample uptake interval. Each analysis consisted of 5 runs, each including 50 sweeps through the mass range. Dwell times were held constant for all analytes (10 ms), leading to a total count time of 1.25 s per analyte. Each analysis was followed by a 60 s washout using ultra-pure 2% HNO3 (*v*/*v*).

The analytical sessions began with approximately one hour of instrument warmup prior to tuning the ICP-MS. Each experiment was calibrated using a mixed standard solution prepared by diluting a multi-element standard solution (CAT# 51844, Lot: BCCC7938; Sigma Aldrich; St. Louis, MO, USA) 2000-fold. Calibration curves were generated for each analytical run from a blank and eight serial dilutions of this mixed standard ranging from 200-fold to undiluted. Instrument drift and matrix effects were monitored by spiking all calibration standards and samples with 0.5 ppb Rh (CAT# CGRHN1; Inorganic Ventures; Christiansburg, VA, USA) and Re (CAT# CGRE1; Inorganic Ventures; Christiansburg, VA, USA). Instrument drift was also monitored and corrected (if necessary) by the re-analysis of a single calibration standard every 25 samples and the entire calibration block at the end of the experiment. External reproducibility and accuracy were assessed through repeat analyses of NIST 1577b (bovine liver) digests showing good agreement with accepted values (Supplementary Figure S1).

The limit of detection (LOD) was calculated as 3 × SD of the blanks, and the limit of quantification (LOQ) was calculated as 10 × SD of the blanks from the average of three analytical sessions (Supplementary File S1).

### 2.6. Collection of Information from Producers

Participating farmers were asked to complete a voluntary questionnaire about diets, supplementation strategies, and the perception of the health status of their flock (Supplementary File S2). Alternatively, they were verbally interviewed using the same questionnaire. We achieved a 100% (*n* = 56) response rate for essential information including animal body condition scores, ages (when known), production stage, and mineral supplementation used. For other questions, we achieved a 43% survey completion rate (*n* = 24).

For survey results, the following equations were used:

$$Sensitivity=True\;Positive\;÷\left(True\;Positive+False\;Negative\right)$$

$$Specificty=True\;Negative\;÷\left(True\;Negative+False\;Positive\right)$$

$$Positive\;Predictive\;Value=True\;Positive\;÷\left(True\;Positive+False\;Positive\right)$$

Negative Predictive Value=True Negative ÷(True Negative+False Negative)

### 2.7. Data Processing and Statistical Analysis

ICP/MS values were calculated in ppb (ng/mL), and values below the LOD were not considered for statistical and descriptive analyses (Supplementary File S1). The comparison between whole blood and plasma values was performed in GraphPad Prism. Outlier detection was performed using ROUT method (Q = 5%) in GraphPad Prism. All figures were generated using GraphPad Prism version 10.3.1 for macOS (GraphPad Software; Boston, MA, USA). The comparison of the microminerals between the two laboratories was performed using PROC GLM of SAS (v9.4), with laboratory or type of sample (plasma or whole blood) as a main effect and sample as a random effect. The Proc CORR of SAS was used to obtain the Pearson correlation and the related *p*-value. The paired comparison of royal blue vs. purple blood tubes was carried out using the Proc TTEST function of SAS.

## 3. Results

### 3.1. Validation of the Whole Blood Micromineral Analysis Method

The mineral values included in the Texas A&M University Veterinary Diagnostic Laboratory (TAMU) trace element panel were compared with those achieved using our method. A robust correlation (r ≥ 0.80), according to the criteria from Evans (1996) [26], was found between labs for Mo and Se (Table 1). Poor agreeability (r < 0.54 and *p* > 0.07) was found between the two labs for Co, Cu, Fe, Mn, and Zn. Besides a poor correlation between the two laboratories, Co and Mn were >3- and >5-fold larger in TAMU vs. OSU (*p* < 0.001), while the values of the other microminerals were not statistically different between the two labs. Our values were repeatedly checked against NIST 1577b (bovine liver) digests and produced good agreement (Supplementary Figure S1).

### 3.2. Sample Summaries

Descriptive sample summaries are outlined in Table 2. In total, 370 sheep were sampled across 56 farms located in Oregon. The mean age of sheep was 3.5 years, and the median body condition score (BCS) was 3.5. The 10th and 90th percentile ranges observed in our data are also reported and were chosen to eliminate outliers while still capturing the overall spread and skewness of the data per farm.

### 3.3. Prevalence of Deficiency or Excess According to Literature Values

Reference values for whole blood concentrations of As, Cd, Pb, and Se in sheep have been clinically established [23,25]. Various literature sources have previously reported reference values for other minerals in whole blood of sheep [25,27].

**Figure 1:**
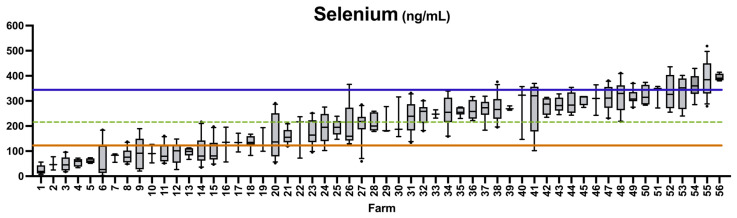
The status of each flock is ranked by mean. The mean level of Se ranks farms. The dashed line indicates the overall mean Se levels in all farms (205 ng/mL). Space between the top and bottom lines captures the normal reference range (120–350 ng/mL). Bars indicate 10th and 90th percentiles.

The mean Se value for all sheep in this study was 205 ng/mL. Farms were ranked by flock mean Se (Figure 1). Between three and thirteen animals were included per farm. In our study, 27.6% (*n* = 102) of sampled sheep were classified as Se-deficient (<120 ng/mL), while 10.5% (*n* = 39) of sheep sampled had Se levels exceeding the normal range (>350 ng/mL) (Table 3). When analyzed at the farm level, 26.8% (*n* = 15) of farms had an overall flock mean Se concentration below the deficiency threshold, while 5.4% (*n* = 3) had a flock mean Se concentration above the normal range (Table 3).

**Figure 2:**
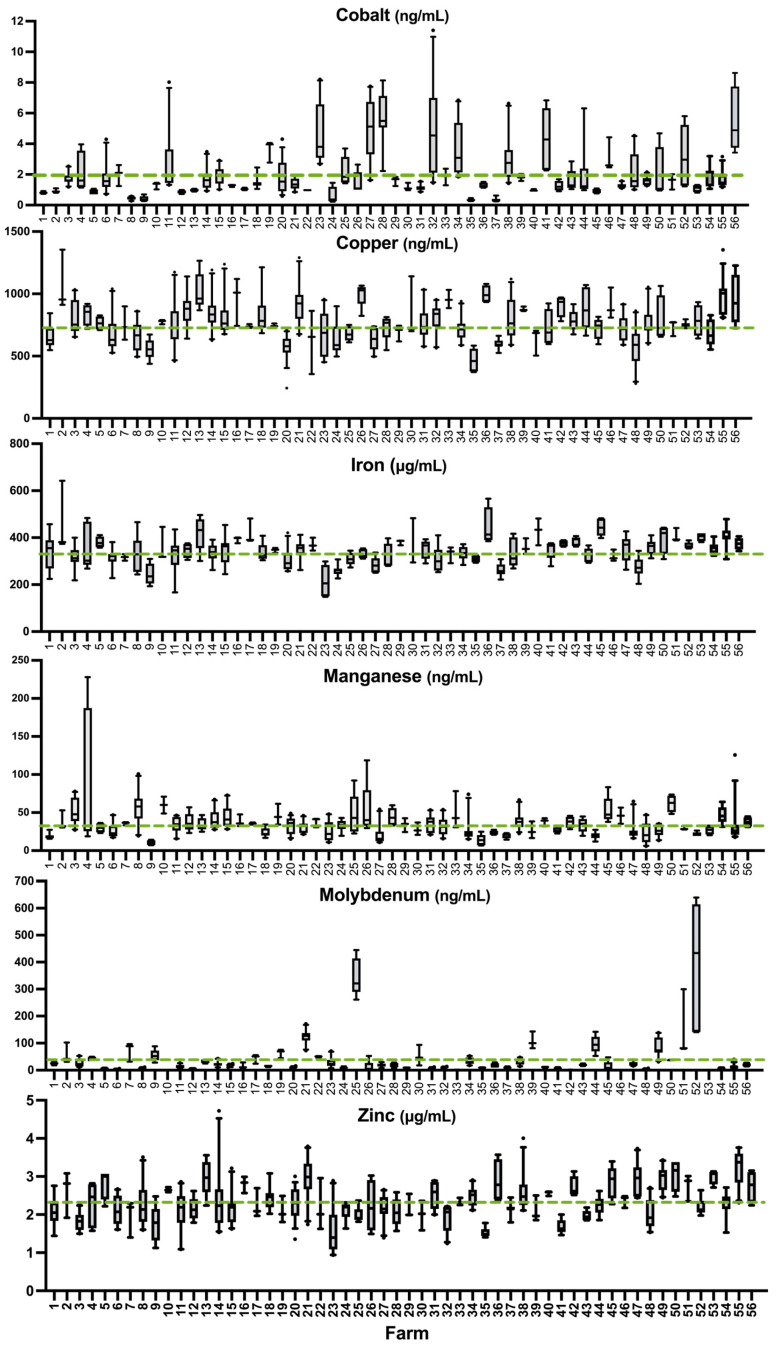
Essential element status in whole blood of all samples above the LOD for essential elements on 56 farms arranged by farm according to mean Se rank. The dashed lines indicate the overall mean of each mineral in all the farms.

Essential trace elements Co, Cu, Fe, Mn, Mo, and Zn, with no known whole blood reference values, are graphically depicted in Figure 2. Mean values of each element in our sample are indicated in Table 2. Two sheep from the same farm (0.54% of the total sheep sampled) had Pb levels above the acceptable limit (>250 ng/mL), whereas no sheep were found to have excess levels of As, Hg, or Cd. More than half of the sheep in this sample were deficient in Cu, Fe, Mn, and Zn (Table 3). Seven individuals from seven different farms were identified as marginally above the higher reference limit for Cu (within 155 ng/mL of the upper threshold). No farms had a mean flock concentration exceeding the established thresholds for sheep for As, Cd, Hg, or Pb (Table 3).

### 3.4. Comparison of Sheep Whole Blood Mineral Values to Established Whole Blood Reference Values

**Figure 3:**
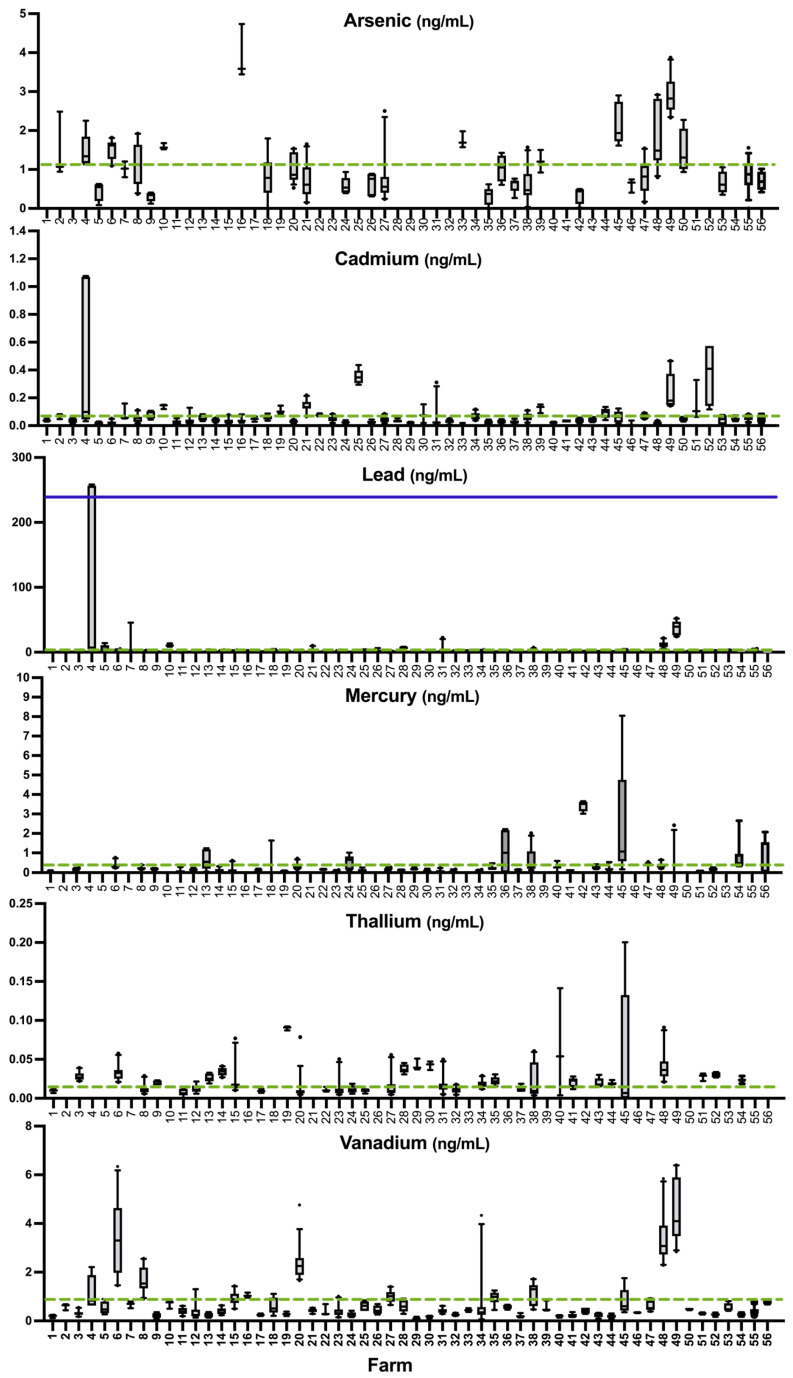
Status of toxic elements in whole blood of all samples above the LOD for each element on 56 farms, ranked by the mean of Se. The dashed line indicates the overall mean of each element by farm. A solid line is included at the maximum reference threshold when samples exceed it.

Farm status of minerals with no known nutritional benefits that are associated with toxicity, including As, Cd, Hg, Pb, Tl, and V, are graphically depicted in Figure 3. Mean values for each element in our sample are reported in Table 2. The lesser-studied minerals Al, B, Ba, Be, and Cr are included in Supplementary Figure S2. Human whole blood threshold limit values for As, B, Ba, Be, Cd, Co, Cr, Hg, Pb, and Tl have been clinically established. For As, B, Be, Cd, Cr, Hg, Pb, and Tl, the majority (59.2–100%) of sheep fell below the maximum threshold limit as set for humans even when it was much lower than the maximum threshold limit set for sheep (e.g., As, Pb) (Table 3 and Table 4). No sheep samples fell within the reported human whole blood threshold limit values for Ba (Table 4). Co concentration in sheep whole blood fell starkly below the literature value provided for sheep by Georgievskii et al. [28] but it is aligned more closely with the reference limit for human whole blood.

**Figure 4:**
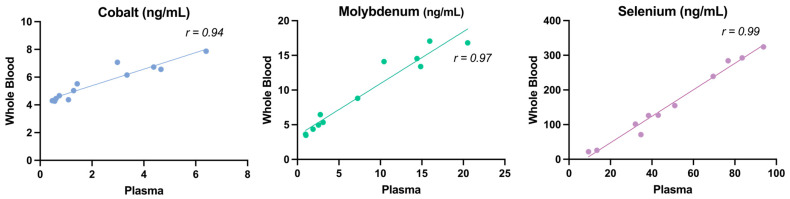
Scatter plots for minerals Co, Mn, and Se where whole blood and plasma values have a strong correlation (r > 0.90).

To assess the feasibility of using whole blood instead of plasma for diagnostic purposes, particularly when centrifugation is unavailable, plasma and whole blood samples were analyzed for several essential trace elements by the TAMU lab, a clinical veterinary diagnostic lab. There was significant agreement between serum and whole blood values for Co, Cu, Fe, Mo, and Se. For Co, Mo, and Se; the correlation was extraordinarily high and allowed us to calculate with high confidence a linear equation to derive the whole blood ranges from the published plasma/serum ranges (Table 5; Figure 4).

### 3.5. Correlation Between Whole Blood Minerals, Age, and Body Condition Score

There were several significant (*p* < 0.05) correlations between whole blood minerals in sheep ranging from weak to strong (r < 0.20 to 0.73) [26] (Supplementary File S3). Moderate but significant positive correlation (0.40 ≤ r ≤ 0.59) was found between Tl and As; Tl and Hg; Al and Be; V and As; and Mn and Pb. A significantly high positive correlation (0.60 ≤ r ≤ 0.79) was found between Mo and Cd; Pb and Cd; and Zn and Fe. There was no discernible correlation between mineral status and age or body condition score of animals.

### 3.6. Survey Results

**Figure 5:**
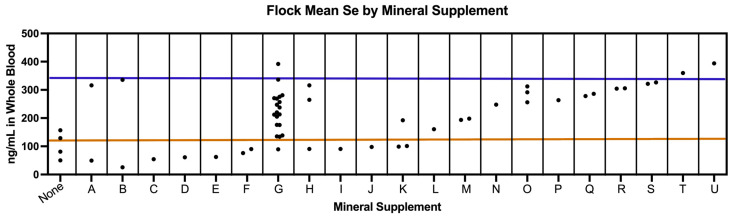
Mineral supplement brands are denoted by a letter (A-U) or none. Each dot represents a farm (*n* = 56). Farms are plotted by flock mean Se (ng/mL in whole blood) and mineral supplement strategy. The area between the solid lines indicates the normal reference range (120–350 ng/mL).

Twenty-two different supplementation strategies were used by the 56 participating farms (Figure 5). There was a substantial difference between inter-flock mean Se. Four farms (#3, #8, #16, #20) reported using no supplement. Two farms (#27 and #56) reported using Se forage fertilization in addition to an ad libitum mineral supplement. One farm reported using a bovine block mineral (#12) and another reported using a multi-mineral bolus (#5). All other farms reported providing an ad libitum loose mineral supplement. None of the producers who completed the survey reported using injectable Se in ewes regularly. Fifteen out of twenty-four producers (62.5%) who completed the survey reported using injectable Se in lambs either routinely or for weak lambs. Out of 42 farmers who indicated their perceived flock Se status, nine perceived their flock to be less than sufficient in Se, of whom four accurately predicted their flock mean was inadequate in whole blood Se (<120 ng/mL) (Supplementary Figure S3). Farms #6, #7, #8, and #10 (*n* = 4) correctly predicted their flock mean was less than sufficient, while farms #19, #23, #45, #49, and #50 (*n* = 5) incorrectly predicted their flock mean was less than sufficient. Farms #3, #4, #5, #11, #13, #14, and #15 (*n* = 7) inaccurately predicted their flock mean Se status was sufficient when it was less than sufficient. Sensitivity was 36.4%, while specificity was 83.9%. The positive predictive value (PPV) for Se deficiency was 44.4% and the negative predictive value (NPV) for Se deficiency was 78.8%.

### 3.7. Comparison Between Mineral Concentrations in Blood Collected into Standard vs. Mineral-Free EDTA Tubes

Blood samples collected from 14 sheep in both blue- and purple-top tubes to evaluate potential mineral contamination from purple tubes yielded no significant difference for any of the 18 minerals tested (*p* > 0.18) (Table 6).

### 3.8. Establishment of Whole Blood Reference Values for 18 Minerals in Oregon Sheep

Per the American Society for Veterinary Clinical Pathology (ASVCP) guidelines, a sample size ≥120 is recommended to establish reference intervals [39]. Friedrichs et al. note that for normally distributed data, reference intervals can be estimated using a nonparametric method, typically encompassing the central 95% of values [39]. Outliers were identified and removed using the Robust Regression and Outlier Removal (ROUT) method, which combines robust regression with a false discovery rate (FDR)-based approach (Q = 5%). A more permissive Q threshold was selected to flag a broader range of atypical values. In our data set, all 18 minerals were normally distributed, and for each mineral, at least 179 animals had values above the limit of detection after removing outliers. We therefore propose reference intervals for each mineral in Oregon sheep, as presented in Table 7, using the central 90% of values in the Oregon sample after the removal of outliers to maintain a stringent estimation framework.

## 4. Discussion

### 4.1. Method Validation

Low and insignificant correlations between lab total element counts across samples are attributed to differences in digestion and measurement methods. In contrast to our method, which digested whole blood prior to analysis, TAMU used a “dilute-and-shoot” (i.e., straight-injection) method. Our digestion protocol, which uses heat, nitric acid, and hydrogen peroxide, would have reduced carbon-related interferences and improved the accuracy of ICP/MS reads [40,41,42,43]. Tissues often require thermal and/or chemical digestion for ICP/MS to solubilize organically bound elements. In our comparison of NIST 1577b bovine liver standard reference material, we observed excellent agreement for all mineral concentrations, including Co and Mn, which supports the digestion method yielding a more accurate result. Despite this methodological difference, we found good agreement between labs for Mo and Se. The values we obtained for Co and Mn agree well with those provided by Ademi et al. (2017) [27] in adult ewes and with NIST 1557b; however, the TAMU straight-injection method resulted in much higher concentrations for these minerals. Sample degradation or volatilization during shipping is unlikely to account for elevated concentrations of any mineral and does not explain why TAMU reported Co and Mn values exceeding ours by more than 3- to 5-fold. While resolving methodological differences is beyond the scope of this study, we note that our digestion protocol shows high agreement with the certified values of standard reference material NIST 1577b, supporting the accuracy of our measurements. Ultimately, we do not believe these inter-lab discrepancies affect the interpretations presented in the manuscript.

### 4.2. Comparison of Whole Blood Values to Known Plasma Reference Values in Sheep and Whole Blood Reference Values in Humans

Clinical reference values for whole blood in sheep have not been established for any minerals except As, Cd, Pb, and Se, despite whole blood being the more practical sample type for testing. In the absence of clinical reference ranges, definitive diagnoses of mineral deficiencies cannot be made. Instead, veterinarians, producers, and nutritionists must rely on literature values, which may vary based on factors such as diet, breed, physiological status, environmental conditions, and management practices. We aimed to establish whole blood reference values for ewes by sampling a diverse cohort of grazing sheep across multiple farms in Oregon. We compared whole blood and plasma mineral concentrations and evaluated whether sheep whole blood values align with established human reference ranges to assess the appropriateness of cross-species comparisons. This approach provided a practical framework for contextualizing sheep mineral values in the absence of extensive species-specific reference data and was intended to expand the foundation for sheep whole blood reference values without requiring the large-scale sampling of healthy animals. Ademi et al. [27] previously used a similar strategy in their evaluation of the sheep population in Kosovo, Norway, demonstrating the utility of human reference intervals as an initial benchmark where direct comparisons within species are limited [27]. Still, species-specific physiological differences may limit direct comparability, and further work is needed to establish definitive reference values for sheep.

The strong correlations observed between whole blood and plasma values for Co, Mo, and Se suggest that whole blood could serve as a reliable alternative when plasma separation via centrifugation is not feasible. The derived linear equations (Table 5) allow for the conversion of whole blood concentrations to plasma-equivalent values, enabling more flexible sample collection in field settings or resource-scarce environments. This is particularly valuable for on-farm monitoring or large-scale epidemiological studies where immediate processing of whole blood is impractical and when whole blood reference values are unestablished. However, further validation across different physiological conditions, breeds, and species may be necessary to refine these equations and ensure accuracy.

The observation that whole blood mineral concentrations of As, Be, Cd, Cr, Hg, Pb, and Tl in sheep largely align with human threshold limit values (TLVs) indicates that these reference values may be used until sheep whole blood reference values can be established for those minerals. However, discrepancies were observed for B and Ba, where sheep whole blood values exceeded human TLVs. This may indicate that the bioaccumulation of these minerals is not comparable between these species and perhaps that the normal physiological concentrations of these elements in sheep may differ from those in humans potentially due to differences in dietary intake, metabolism, or excretion mechanisms. While human TLVs provide a preliminary reference point, considering the lack of reference ranges for ruminants, direct comparisons should be interpreted cautiously. As production animals, sheep have distinctly different physiological requirements and tolerable levels for certain minerals [27,44]. Therefore, what is considered elevated in humans may not necessarily indicate risk in sheep. Importantly, none of the sheep in this study experienced symptoms of B or Ba toxicity [25]. Still, establishing species-specific whole blood reference values is essential for accurately assessing mineral status and potential toxic exposure in sheep. Doing so would enhance diagnostic capabilities and expand access to mineral deficiency and toxicity screening for producers.

### 4.3. Trace Mineral-Free Blood Tubes

In our study, there was no significant difference between mineral values when blood was collected from the same animal into a royal blue-top mineral-free EDTA tube compared to a purple-top EDTA tube. This result suggests that when royal blue mineral-free tubes cannot be sourced or are cost-prohibitive (a 46% price difference at the time of this study; Tiger Medical, Wayne, NJ, USA), a purple top vacutainer tube may be an appropriate alternative when used to measure the minerals in our panel. Due to variability between batches and other manufacturing factors, if a purple tube is used and elevated mineral levels are detected, results should be confirmed by resubmitting blood in a royal blue trace mineral-free tube.

### 4.4. Prevalence of Selenium Deficiency

As part of the pre-selection process, producers were asked if they had concerns about mineral deficiencies in their flocks. While nine farmers predicted that their flocks were less than sufficient in Se status, none reported concerns about symptomatic Se deficiency, neither in conversation nor indicated in the survey. Yet, 27% of farms (*n* = 15) had a mean flock Se status below the deficiency threshold. Surprisingly, none of these farmers reported experiencing health or production deficits, suggesting that while Se levels were deficient, the effects were likely subclinical in adult ewes. This is consistent with known clinical presentation of Se deficiency in humans, sheep, and other ruminants, where adults generally do not show overt symptoms, particularly in the absence of complicating stressors [10,11,12,13,14,15,16]. The positive and negative predictive values indicated that while farmers were fairly good at predicting sufficient flocks (high specificity and NPV), their ability to correctly predict inadequate flock stats was lower (low sensitivity and PPV). In sheep, symptoms of Se deficiency include myodegeneration (white muscle disease), reduced reproductive performance, and impaired immune responses [25]. White muscle disease typically affects young, rapidly growing lambs that are deficient in Se. However, while closely linked to Se levels, the disease is more broadly attributed to inadequate antioxidant status rather than Se deficiency alone [45,46,47,48,49]. However, it is a rare disease in adult animals. This aligns with the understanding that Se deficiency becomes clinically apparent primarily when it compromises antioxidant defenses [45,46,47,48,49,50,51,52,53,54,55,56]. Most functional effects of Se deficiency, such as WMD and immune dysfunction, are mediated through impaired selenoprotein activity. Therefore, deficiency may persist without visible signs unless antioxidant demand increases beyond what can be compensated. Furthermore, the observation of impaired immune responses depends on exposure to immune challenges that may not have occurred or been noticed in our study flocks, especially those that are closed and well-managed. Reproductive performance is impacted by many factors and can be artificially manipulated (e.g., flushing, estrus synchronization). Importantly, our study population consisted of healthy adult ewes without recent illness and who were previously able to maintain pregnancy; animals showing signs of poor health were excluded from sampling. This likely contributed to the lack of observable clinical signs of deficiency or toxicity. Our findings further support that relying on symptoms in adult sheep is an unreliable means to detect Se deficiency in adults, and evaluating the efficacy of a diet and supplementation regimen is essential.

Our inclusion criteria were designed to select animals representative of the broader Oregon sheep population with respect to management practices, production stage, and overall health. The National Agricultural Statistics Service reports that as of 2025, 78% of all breeding sheep and 57% of all sheep in the U.S. are ewes older than one year of age [57]. By focusing on healthy, dry ewes, our survey captures the mineral status of the most common demographic within the nation, although the exclusion of unhealthy animals means our findings primarily reflect the mineral status of well-managed, pasture-fed operations rather than perhaps the entire population of Oregon sheep. The data provide an important reference point for mineral concentrations in healthy, dry ewes managed under typical Oregon conditions during our sampling months.

One of the most surprising results in our study was the prevalence of deficiency despite farmers reporting providing Se supplementation. In contrast, Ademi et al. [27] found a negative relationship between Se supplementation and deficiency rate. The substantial variation in mean Se values among flocks using the same supplement may be influenced by differences in forage Se content, the frequency of mineral replenishment, or factors affecting the supplement’s accessibility and palatability such as placement. Farmers should be encouraged to ensure that flocks are consuming ad libitum mineral supplements at a rate compatible with the recommended feeding guideline and to routinely test Se blood levels in the flock to screen for deficiency. In areas with endemic Se deficiency, Se-fortified forages have emerged as a promising solution [8,44,58,59]. While factors like weather spoilage and palatability can negatively influence flock consumption of ad libitum mineral supplements, Se fortification circumvents this issue altogether by providing Se ubiquitously in the pasture-based diet. Additionally there is mounting evidence that selenomethionine—the primary Se species found in Se-fortified forages and known for its high bioavailability—has a unique role in Se metabolism and antioxidant defense, distinct from that of inorganic forms such as selenite or selenate [58,59,60,61,62,63,64,65]. Therefore, future research into the nutritional benefits of Se should not only consider total Se but aim to delineate the specific contributions and metabolic fates of individual Se species.

### 4.5. Prevalence of Deficiency of Other Essential Trace Elements

#### 4.5.1. Cobalt

The ovine whole blood reference value provided in the literature (30–50 ng/mL) would suggest that all individuals in our study were deficient in Co [28]. Our study and that of Ademi et al. [27] had a sample mean whole blood Co concentration of 2.0 and 1.4 ng/mL, respectively, and all animals in these samples would be considered to have an inadequate level of Co. In neither study did animals exhibit symptoms of Co deficiency, and in both studies, the range of Co levels better aligned with the human clinical reference (<1.8 ng/mL) or the sheep plasma reference range (0.18–2.0 ng/mL) rather than the whole blood reference range provided in the literature. This study, along with our previous work, provides further evidence that previously established whole blood Co levels may poorly represent the normal range in domestic sheep populations [27,64,65].

#### 4.5.2. Copper

While 64% (*n* = 240) of our sample was considered copper-deficient, these values should be validated with liver values. No farms were identified as having a mean whole blood Cu concentration above the toxicity threshold, not even one farm that reported using copper oxide wire boluses (#43). Ovine liver tissue has a high affinity for Cu and tends to sequester it; blood levels of Cu typically rise only when the liver becomes so overloaded that it suddenly releases Cu into the blood causing hemolysis [23,66,67]. Thus, blood Cu is regarded as a non-definitive test for diagnosing Cu status in sheep [23,44,67]. Because of the hazard of Cu toxicity and how impractical liver toxicity screening is, many multi-mineral supplements omit Cu entirely, which may position animals for chronic deficiency if the diet is inadequate. Producers should consider postmortem liver sampling as a practical and more accurate means of gauging flock Cu levels to determine if deficiency is prevalent.

#### 4.5.3. Molybdenum

Mo is an essential trace element that plays a particularly unique role in sheep, wherein it has been known to interact with Cu to prevent Cu toxicity [25]. While a whole blood reference value has not been established for sheep, our study found a strong correlation between plasma and whole blood concentration for Mo, which allowed us to estimate the normal range for whole blood to be between 4.2 and 39 ng/mL, and 71% of individuals in our sample (*n* = 256) fell within this expected range. Molybdenum levels varied widely across farms but had little variability between individual animals on each farm except for a few farms (#25, #51, and #52). Sheep in this study and as described in the literature seem to have relatively high tolerance for Mo, and tolerance is influenced by the chemical form of Mo, type of diet, sulfur in the diet, and Cu status [44]. Deficiency symptoms are poorly described in the literature.

#### 4.5.4. Iron

There was a relatively high prevalence of Fe deficiency, which, in ruminants, is likely caused by intestinal hemorrhage secondary to helminth infection rather than Fe-deficient diet [25,68,69]. Whole blood testing alone does not clarify whether the observed Fe deficiency in sheep is a primary nutritional deficiency or secondary to blood loss. While we selected for well-appearing ewes, obvious symptoms of parasitosis (e.g., loss of body condition and scours) develop after persistent exposure or rapid burden are preceded by decreased Fe secondary to blood loss anemia [68,69,70]. Future research should explore the relationship between dietary Fe intake, whole blood Fe, hematocrit, fecal occult blood, and parasitic burden to assess their potential as early indicators of parasitic infection, either as an alternative or complement to fecal egg counts. Such work could enhance our ability to detect subclinical parasitism and guide early interventions. Taken together, these findings highlight the need for further research to establish clear reference intervals for whole blood Fe and related biomarkers in sheep.

#### 4.5.5. Manganese

Our samples had a similar range of Mn in the whole blood of ewes (5.9–228 ng/mL) as the study by Ademi et al. [27] (6.0–243 ng/mL). When using the literature referenced by Ademi et al. [27] of 70–200 ng/mL, both studies resulted in a high (>96%) inadequacy rate. However, the whole blood reference range provided by Puls [25] of 20–40 ng/mL would indicate an inadequacy rate of 19% and an adequacy rate of at least 52.5% in our sheep samples. Symptoms of toxicity or deficiency of Mn are poorly characterized in mature sheep, and despite being an essential element, a wide range of Mn statuses appears to be well-tolerated [25,44]. For example, the threshold for toxicity in ppm of the diet is 30- to 80-fold higher than that of the adequate level [25]. Our results indicate that the reference range for whole blood Mn in sheep, as provided by Puls [25], is applicable. However, this range may warrant further expansion given the absence of Mn deficiency symptoms in the sheep we surveyed and the wide degree of tolerance described in the literature [25,44].

#### 4.5.6. Zinc

According to the literature values, there was a high rate of Zn inadequacy in the sheep we surveyed, but it is unlikely the animals truly experienced nutritional Zn deficiency. The range of Zn detected in our study (0.94–4.72 μg/mL) was similar to the one reported by Ademi et al. [27] (1.4–3.8 μg/mL). Symptoms of Zn deficiency include anorexia, skeletal and reproductive disorders, impairment of the immune system, weak hoof walls, and poor skin and wool [25,44], which were not observed in the surveyed sheep. Owing to the fact that no Zn retention pool has been identified in any mammal, whole blood and other bodily tissues are not ideal matrices for diagnosing adequacy or deficiency [25,71]. Instead, Zn homeostasis appears to be controlled by changes in gastrointestinal absorption and excretion [72]. Serum and plasma are the preferred matrices for maximizing accuracy in diagnosing Zn status but are not without limitation; age, sex, time of day, recent dietary intake, stress, illness, and a plethora of other intrinsic factors can influence Zn levels [73]. Alternative tests have been proposed to improve accuracy in Zn status diagnosis, such as measuring hair Zn accumulation or measuring the abundance of linoleic acid relative to dihomo-γ-linoleic acid (DGLA), as the conversion of linoleic acid to DGLA is a Zn-dependent process [74]. Given these complexities, relying solely on whole blood Zn concentrations may lead to the misclassification of deficiency [75]. Until improved tests become available, maintaining adequate Zn in the diet of sheep and testing plasma or serum when Zn deficiency is suspected remains the accepted recommendation.

### 4.6. Other Potentially Essential or Toxic Elements in Sheep Whole Blood

The nutritive value of Al, As, B, Ba, Be, Cd, Cr, Hg, Pb, Tl, and V remains unestablished in sheep and several of these minerals are associated with toxicity [25,38,44,76,77,78,79]. All of these minerals exist at low levels in U.S. soil under normal circumstances [25,80]. For Al, B, Ba, Be, Cr, Tl, and V, a whole blood threshold in sheep has not been established, which poses a challenge for interpreting the clinical relevance of our results; however, none of the sheep were suspected of any mineral toxicity. While maximum tolerable levels in the diet of ruminants have been described in the literature, an association between toxic dietary levels and whole blood accumulation has not been made [25,44]. It would be highly beneficial to establish tolerable levels of potentially essential or toxic elements in the whole blood of sheep. However, careful consideration must be given to the specific form and metabolized state of each element, as not all forms pose the same risk of toxicity (e.g., Al, Ba, Hg) [25,81].

### 4.7. Proposed Whole Blood Reference Values for 18 Minerals

The utility of the reference intervals proposed here depends on the assumption that the sampled population is broadly representative of healthy adult sheep. For example, while the 5th–95th percentile range for Se in our population was 34–380 ng/mL, values below 120 ng/mL may still indicate marginal or subclinical deficiency. Normal clinical reference intervals for Se in the whole blood of sheep typically begin at 120 ng/mL, which is informed by case reports of animals suffering from Se deficiency-like symptoms at blood levels <120 ng/mL [23]. Notably, in 2011, Herdt and Hoff explicitly stated that reference ranges generated from means of populations of known healthy herds are not available [23]. Our study is one of few to address this knowledge gap. Still, we interpret values below 120 ng/mL with caution despite their inclusion in the statistical reference interval. Similar caveats apply to other minerals, such as Cu, Zn, and Mn, where values within the statistical reference range may not reflect biological adequacy, and with toxic elements where the tolerance threshold is much higher than the maximum in our data set [23,25]. Although our study includes several cases where animals below the Se deficiency threshold showed no apparent health or production deficit based on owner reports, we emphasize that the preliminary reference intervals derived from this limited study do not necessarily distinguish between physiologically sufficient and insufficient levels. These proposed values should be interpreted in conjunction with clinical findings, dietary intake, production stage, and functional indicators when available. Further research including replication studies is needed to validate the reference ranges proposed here. It is our intention that this dataset may contribute to broader efforts, including future meta-analyses or multi-regional surveys of healthy flocks to establish more robust and globally relevant normal reference intervals for whole blood mineral status in sheep.

### 4.8. Limitations

Although whole blood presents as a more preferred biological sample to submit for testing compared to liver biopsy or plasma, ovine whole blood reference ranges remain unestablished for several microminerals. While more convenient, the biological relevance of whole blood value is questionable for a few microminerals (e.g., copper, cobalt), and hepatic concentrations are more sensitive to detect toxicity or deficiency [23]. All mineral supplementation data from producers were self-reported and could not be validated. As such, while producers may have reported providing a supplement ad libitum, it is probable that some flocks did not have consistent access to their mineral supplement in the period leading up to blood collection. Survey data regarding flock diet was ultimately not presented because little inference could be made without forage mineral testing. Future studies should aim to corroborate forage mineral levels, especially in areas with assumed endemic deficiency.

## 5. Conclusions

Despite several farms reportedly using a similar supplementation strategy, there was considerable variability between mean Se status. Confidence in the supplementation protocol should be reinforced by testing individuals’ mineral levels to ensure effectiveness, particularly since no owners reported observing symptoms of deficiency in their animals. We demonstrated the feasibility of using the whole blood matrix and standard EDTA K2 purple top tubes as an alternative to plasma collected from trace mineral-free royal blue tubes, which may make testing more accessible to producers. Our findings add to the growing body of reference data on the levels of 18 different elements in the whole blood of grazing sheep. When ovine whole blood clinical reference ranges are unestablished, those available for ovine plasma (Co, Cu, Fe, Mn) or human whole blood (As, B, Be, Cd, Cr, Hg, Pb, and Tl) demonstrate good agreeability with the range of our samples. Mineral status does not appear to be influenced by age nor body condition score of individuals. Future research should aim to establish clinical reference values for ovine whole blood mineral concentration and corroborate them with other matrices, such as liver, kidney, and plasma. Additionally, a deeper understanding of existing reference values is needed to determine the practical significance of subclinical deficiencies.

## Figures and Tables

**Table 1:** Comparison between microminerals analyzed in the whole blood of 12 sheep in 4 farms by WM Keck Collaboratory for Plasma Spectrometry at Oregon State University (OSU) and the Texas A&M University Veterinary Diagnostic Lab (TAMU) diagnostic laboratory.

Mineral	Unit	OSU	TAMU	SEM	*p*-Value	r	*p*-Value ^1^
Co	ppb	1.74	5.58	0.30	<0.001	0.45	0.14
Cu	ppb	753	823	35.2	0.17	0.19	0.54
Fe	ppm	332	339	15.6	0.75	0.20	0.53
Mn	ppb	31.1	182.2	5.57	<0.001	0.53	0.08
Mo	ppb	11.3	9.41	1.77	0.45	0.85	<0.001
Se	ppb	153.8	170.3	30.2	0.70	0.98	<0.001
Zn	ppm	2.13	2.33	1.35	0.30	0.46	0.13

^1^ *p*-value of r.

**Table 2:** Sample summaries.

					Percentile					
Mineral	Units	*n* ^1^	Min	Max	25th	50th	75th	90th	Mean	SD
Age	years	284	2	7	3	3	4.2	5	3.5	1.17
BCS ^3^		370	2.0	5.0	3.0	3.5	4.0	5.0	3.3	0.54
Co	ng/mL	370	0.23	11.41	1.05	1.43	2.37	4.70	2.0	1.72
Cu	ng/mL	370	244	1355	641	739	868	1004	762	176
Fe	µg/mL	370	148	642	299	340	377	412	339	65
Mn	ng/mL	370	5.9	228	22.0	31.2	41.7	54.3	34.6	20.3
Mo	ng/mL	363	0.132	640	8.4	16.2	34.1	87.2	38.3	74.2
Se	ng/mL	370	2.1	519	105	213	289	354	205	113
Zn	µg/mL	370	0.94	4.72	1.97	2.29	2.67	3.07	2.34	0.57
Minerals with Uncertain Dietary Requirement ^2^										
Al	ng/mL	370	138	4897	645	960	1317	1841	1063	585
As	ng/mL	198	0.084	4.74	0.59	0.902	1.50	2.29	1.1	0.81
B	ng/mL	370	30	701	125	171	255	333	201	119
Ba	ng/mL	370	13.5	888	74	118	204	324	164	137
Be	ng/mL	280	0.001	0.206	0.023	0.046	0.078	0.106	0.055	0.04
Cd	ng/mL	367	0.001	1.076	0.025	0.041	0.069	0.131	0.070	0.11
Cr	ng/mL	370	3.23	100	13.2	25.3	26.6	28.7	21.4	10.6
Hg	ng/mL	273	0.01	8.05	0.10	0.17	0.28	0.64	0.36	0.71
Pb	ng/mL	370	0.29	258	1.00	1.51	2.88	5.38	4.80	19.8
Tl	ng/mL	261	0.002	0.200	0.010	0.017	0.029	0.040	0.022	0.02
V	ng/mL	370	0.052	6.41	0.28	0.45	0.89	2.26	0.87	1.10

^1^ Number of samples above the detectable limit as determined by the LOD; age when known. ^2^ Elements with no established dietary requirement, including those with potential toxicity and those with unclear physiological roles. ^3^ Body condition score.

**Table 3:** Number and percent of surveyed sheep and farms (mean concentration of the element) above or below the whole blood values for minerals according to clinical and literature references.

						Adequacy		
Mineral	Reference	Units	Reference	# Above	# Below	% Above	% Below	% Within
Sheep (*n* = 370)								
Co	30–50	ng/mL	[28]	0	370	0	100	0
Cu	800–1200	ng/mL	[27]	7	238	2.0	64.0	34.0
Fe	360–420	µg/mL	[27]	31	223	8.4	60.2	31.4
Mn	20–40	ng/mL	[25]	106	70	28.6	18.9	52.5
Se	120–350	ng/mL	[23]	39	102	10.5	27.6	61.9
Zn	2.5–6.0	µg/mL	[27]	0	243	0	66.0	34.0
As	<80	ng/mL	[25]	0	NA	0	NA	100
Cd	<200	ng/mL	[25]	0	NA	0	NA	100
Hg	<100	ng/mL	[25]	0	NA	0	NA	100
Pb	<250	ng/mL	[25]	2	NA	0.54	NA	99.5
Farms (*n* = 56)								
Co	30–50	ng/mL	[28]	0	56	0	100	0
Cu	800–1200	ng/mL	[27]	0	35	0	62.5	37.5
Fe	360–420	µg/mL	[27]	5	34	9.0	61.0	30.0
Mn	20–40	ng/mL	[25]	15	5	27	9.0	64.0
Se	120–350	ng/mL	[23]	3	15	5.4	26.8	67.9
Zn	2.5–6.0	µg/mL	[27]	0	0	0	66.0	34.0
As	<80	ng/mL	[25]	0	NA	0	NA	100
Cd	<200	ng/mL	[25]	0	NA	0	NA	100
Hg	<100	ng/mL	[25]	0	NA	0	NA	100
Pb	<250	ng/mL	[25]	0	NA	0	NA	100

**Table 4:** Comparative evaluation of adult human reference ranges for minerals in sheep in whole blood (ng/mL).

				Sample vs. Human TLV ^4^		
Mineral	Sheep Values ^1^	Human Values ^2^	Source	# Above	# Below	% Within
As	<80	<23 ^1^	[29] ^3^	0	370	100
B	-	<200	[30]	151	219	59.2
Ba	-	<5.0 ^1^	[31] ^3^	370	0	0
Be	-	<1.0 ^1^	[32] ^3^	0	370	100
Cd	<200	<5.0 ^1^	[33] ^3^	0	370	100
Co	30–50	<1.8	[34] ^3^	240	130	35.0
Cr	-	<30.0	[35]	25	345	93.2
Hg	<100	<14.9 ^1^	[36] ^3^	0	370	100
Pb	<250	<34.0 ^1^	[37] ^3^	9	370	97.6
Tl	-	<2.0	[38]	0	370	100

^1^ Sheep reference ranges sources reported in Table 3. ^2^ Threshold limit value according to the American Conference of Governmental Industrial Hygienists (ACGIH). ^3^ Human whole blood reference intervals for As, Ba, Be, Cd, Co, Hg, and Pb were obtained from LabCorp and Quest Diagnostics, clinical diagnostic laboratories that derive reference ranges from internal patient data and/or literature sources, though exact sources are not disclosed [29,31,32,33,34,36,37]. ^4^ Sheep in sample (*n* = 370).

**Table 5:** A comparison between microminerals analyzed by the Texas A&M University Veterinary Diagnostic Lab in plasma and whole blood (WB) was made using data collected from 12 sheep from 4 farms.

Mineral	Range ^1^	Plasma	WB	SEM	*p*-Value	R ^2^	*p*-Value ^2^	Formula ^3^
Co	0.18–2.0	2.25	5.58	0.46	<0.001	0.90	<0.001	y = 0.5964x + 4.192
Cu	750–1700	0.93	0.82	0.05	0.11	0.45	0.017	NA
Fe	900–2700	253	34,474	838	<0.001	0.52	0.01	NA
Mn	1.0–6.0	3.30	182.4	5.27	<0.001	0.11	0.30	NA
Mo	1.0–50	7.97	9.15	1.75	0.64	0.94	<0.001	y = 0.7492x + 3.438
Se	60–200	49.6	170.5	24.3	0.002	0.99	<0.001	y = 3.821x − 28.81
Zn	550–1200	0.66	2.33	0.1	<0.001	0.31	0.06	NA

^1^ Normal range of each micromineral in plasma for adult ovine animals reported in ng/mL [23]. ^2^ Pearson correlation between data obtained in plasma and whole blood and related significance. ^3^ Derivatization formula to obtain the value of the reference ranges for micromineral in whole blood using the reference ranges in plasma when the r > 0.90.

**Table 6:** Evaluation of whole blood mineral content in standard (purple) vs. trace-free (royal blue) EDTA tubes.

Mineral	Units	Blue	Purple	SEM	*p*-Value
Al	ng/mL	1161	1081	519	0.58
As	ng/mL	0.81	0.74	0.18	0.71
B	ng/mL	104.7	96.6	5.78	0.18
Ba	ng/mL	104.1	106.0	6.12	0.76
Be	ng/mL	0.07	0.05	0.01	0.32
Cd	ng/mL	0.04	0.04	0.01	0.85
Co	ng/mL	1.67	1.57	0.15	0.52
Cr	ng/mL	7.63	7.48	1.50	0.92
Cu	ng/mL	606	595	23.3	0.66
Fe	µg/mL	279	273	14.7	0.67
Hg	ng/mL	0.19	0.25	0.04	0.16
Mn	ng/mL	29.0	29.0	1.58	0.99
Mo	ng/mL	12.0	12.0	0.84	0.92
Pb	ng/mL	0.80	0.81	0.05	0.85
Se	ng/mL	172.3	164.4	12.6	0.54
Tl	ng/mL	0.02	0.02	0.01	0.46
V	ng/mL	0.85	0.98	0.11	0.24
Zn	ng/mL	2087	2080	112	0.95

**Table 7:** Proposed whole blood reference ranges for sheep according to the Oregon sample.

Mineral	*n*	Range ^1^	Units
Al	362	382–2050	ng/mL
As	179	0.27–1.8	ng/mL
B	357	51–357	ng/mL
Ba	338	32–298	ng/mL
Be	277	<0.120	ng/mL
Cd	320	0.011–0.088	ng/mL
Co	323	0.311–3.17	ng/mL
Cr	367	5.7–30.5	ng/mL
Cu	368	511–1052	ng/mL
Fe	368	244–435	µg/mL
Hg	235	<0.352	ng/mL
Mn	360	14.2–60	ng/mL
Mo	302	3.0–41.7	ng/mL
Pb	322	<3.32	ng/mL
Se	370	34–380	ng/mL
Tl	243	<0.039	ng/mL
V	301	<0.980	ng/mL
Zn	369	1494–3358	ng/mL

^1^ Normative range of each mineral in whole blood for adult ovine animals based on the central 90% of values in the Oregon sample after removing outliers.

## Data Availability

The authors confirm that the data supporting the findings of this study are available within the article and its Supplementary Materials, with producer and animal identifiers removed to protect confidentiality.

## Supplementary Materials

The following supporting information can be downloaded at https://www.mdpi.com/article/10.3390/ani15121799/s1. Supplementary File S1: Raw Whole Blood Minerals Above Detectable Limit; Supplementary File S2: Questionnaire; Supplementary File S3: Correlation Matrix; Supplementary Figure S1: Comparison of Repeat Measurements to Accepted Values of NIST 1577b; Supplementary Figure S2: Levels of al, B, Ba, Be and Cr in Whole Blood; Supplementary Figure S3: Farmers’ Response to Perceived Se Status.
