# Correction: Hasan et al. Status of Selenium and Other Essential and Toxic Elements in Oregon Grazing Sheep. *Animals* 2025, *15*, 1799

**DOI:** 10.3390/ani15233402

**Published:** 2025-11-25

**Authors:** Daniella Hasan, Christopher J. Russo, Katherine R. McLaughlin, Gene Pirelli, Massimo Bionaz

**Affiliations:** 1 Department of Animal and Rangeland Sciences, Oregon State University, Corvallis, OR 97331, USA; 2 College of Earth, Ocean and Atmospheric Sciences, Oregon State University, Corvallis, OR 97331, USA; 3 Department of Statistics, Oregon State University, Corvallis, OR 97331, USA

Text Correction (1 of 9)

There was an error in the original publication [1]. The wrong in-text citations were referenced.

A correction has been made to Discussion, Method Validation, Paragraph 1:

Low and insignificant correlations between lab total element counts across samples are attributed to differences in digestion and measurement methods. In contrast to our method, which digested whole blood prior to analysis, TAMU used a “dilute-and-shoot” (i.e., straight-injection) method. Our digestion protocol, which uses heat, nitric acid, and hydrogen peroxide, would have reduced carbon-related interferences and improved the accuracy of ICP/MS reads [40–43]. Tissues often require thermal and/or chemical digestion for ICP/MS to solubilize organically bound elements. In our comparison of NIST 1577b bovine liver standard reference material, we observed excellent agreement for all mineral concentrations, including Co and Mn, which supports the digestion method yielding a more accurate result. Despite this methodological difference, we found good agreement between labs for Mo and Se. The values we obtained for Co and Mn agree well with those provided by Ademi et al. (2017) [27] in adult ewes and with NIST 1557b; however, the TAMU straight-injection method resulted in much higher concentrations for these minerals. Sample degradation or volatilization during shipping is unlikely to account for elevated concentrations of any mineral and does not explain why TAMU reported Co and Mn values exceeding ours by more than 3- to 5-fold. While resolving methodological differences is beyond the scope of this study, we note that our digestion protocol shows high agreement with the certified values of standard reference material NIST 1577b, supporting the accuracy of our measurements. Ultimately, we do not believe these inter-lab discrepancies affect the interpretations presented in the manuscript.

Text Correction (2 and 3 of 9)

There was an error in the original publication [1]. The wrong in-text citations were referenced.

A correction has been made to Discussion, Prevalence of Selenium Deficiency, Paragraph 1:

As part of the pre-selection process, producers were asked if they had concerns about mineral deficiencies in their flocks. While nine farmers predicted that their flocks were less than sufficient in Se status, none reported concerns about symptomatic Se deficiency, neither in conversation nor indicated in the survey. Yet, 27% of farms (*n* = 15) had a mean flock Se status below the deficiency threshold. Surprisingly, none of these farmers reported experiencing health or production deficits, suggesting that while Se levels were deficient, the effects were likely subclinical in adult ewes. This is consistent with known clinical presentation of Se deficiency in humans, sheep, and other ruminants, where adults generally do not show overt symptoms, particularly in the absence of complicating stressors [10–16]. The positive and negative predictive values indicated that while farmers were fairly good at predicting sufficient flocks (high specificity and NPV), their ability to correctly predict inadequate flock stats was lower (low sensitivity and PPV). In sheep, symptoms of Se deficiency include myodegeneration (white muscle disease), reduced reproductive performance, and impaired immune responses [25]. White muscle disease typically affects young, rapidly growing lambs that are deficient in Se. However, while closely linked to Se levels, the disease is more broadly attributed to inadequate antioxidant status rather than Se deficiency alone [45–49]. However, it is a rare disease in adult animals. This aligns with the understanding that Se deficiency becomes clinically apparent primarily when it compromises antioxidant defenses [45–56].

Text Correction (4 of 9)

There was an error in the original publication [1]. The wrong in-text citations were referenced.

A correction has been made to Discussion, Prevalence of Selenium Deficiency, Paragraph 3:

One of the most surprising results in our study was the prevalence of deficiency despite farmers reporting providing Se supplementation. In contrast, Ademi et al. [27] found a negative relationship between Se supplementation and deficiency rate. The substantial variation in mean Se values among flocks using the same supplement may be influenced by differences in forage Se content, the frequency of mineral replenishment, or factors affecting the supplement’s accessibility and palatability such as placement. Farmers should be encouraged to ensure that flocks are consuming ad libitum mineral supplements at a rate compatible with the recommended feeding guideline and to routinely test Se blood levels in the flock to screen for deficiency. In areas with endemic Se deficiency, Se-fortified forages have emerged as a promising solution [8,44,58,59]. While factors like weather spoilage and palatability can negatively influence flock consumption of ad libitum mineral supplements, Se fortification circumvents this issue altogether by providing Se ubiquitously in the pasture-based diet. Additionally there is mounting evidence that selenomethionine—the primary Se species found in Se-fortified forages and known for its high bioavailability—has a unique role in Se metabolism and antioxidant defense, distinct from that of inorganic forms such as selenite or selenate [58–65]. Therefore, future research into the nutritional benefits of Se should not only consider total Se but aim to delineate the specific contributions and metabolic fates of individual Se species.

Text Correction (5 of 9)

There was an error in the original publication [1]. The wrong in-text citations were referenced.

A correction has been made to Discussion, Prevalence of Deficiency of Other Essential Trace Elements (4.5.1. Copper), Paragraph 1: The ovine whole blood reference value provided in the literature (30–50 ng/mL) would suggest that all individuals in our study were deficient in Co [28]. Our study and that of Ademi et al. [27] had a sample mean whole blood Co concentration of 2.0 and 1.4 ng/mL, respectively, and all animals in these samples would be considered to have an inadequate level of Co. In neither study did animals exhibit symptoms of Co deficiency, and in both studies, the range of Co levels better aligned with the human clinical reference (<1.8 ng/mL) or the sheep plasma reference range (0.18–2.0 ng/mL) rather than the whole blood reference range provided in the literature. This study, along with our previous work, provides further evidence that previously established whole blood Co levels may poorly represent the normal range in domestic sheep populations [27,64,65].

A correction has been made to Discussion, Prevalence of Deficiency of Other Essential Trace Elements *(*4.5.2. Copper), Paragraph 1:

While 64% (*n* = 240) of our sample was considered copper-deficient, these values should be validated with liver values. No farms were identified as having a mean whole blood Cu concentration above the toxicity threshold, not even one farm that reported using copper oxide wire boluses (#43). Ovine liver tissue has a high affinity for Cu and tends to sequester it; blood levels of Cu typically rise only when the liver becomes so overloaded that it suddenly releases Cu into the blood causing hemolysis [23,66,67]. Thus, blood Cu is regarded as a non-definitive test for diagnosing Cu status in sheep [23,44,67]. Because of the hazard of Cu toxicity and how impractical liver toxicity screening is, many multi-mineral supplements omit Cu entirely, which may position animals for chronic deficiency if the diet is inadequate. Producers should consider postmortem liver sampling as a practical and more accurate means of gauging flock Cu levels to determine if deficiency is prevalent.

Text Correction (6 of 9)

There was an error in the original publication [1]. The wrong in-text citations were referenced.

A correction has been made to Discussion, Prevalence of Deficiency of Other Essential Trace Elements *(*4.5.4. Iron), Paragraph 1:

There was a relatively high prevalence of Fe deficiency, which, in ruminants, is likely caused by intestinal hemorrhage secondary to helminth infection rather than Fe-deficient diet [25,68,69]. Whole blood testing alone does not clarify whether the observed Fe deficiency in sheep is a primary nutritional deficiency or secondary to blood loss. While we selected for well-appearing ewes, obvious symptoms of parasitosis (e.g., loss of body condition and scours) develop after persistent exposure or rapid burden are preceded by decreased Fe secondary to blood loss anemia [68–70]. Future research should explore the relationship between dietary Fe intake, whole blood Fe, hematocrit, fecal occult blood, and parasitic burden to assess their potential as early indicators of parasitic infection, either as an alternative or complement to fecal egg counts. Such work could enhance our ability to detect subclinical parasitism and guide early interventions. Taken together, these findings highlight the need for further research to establish clear reference intervals for whole blood Fe and related biomarkers in sheep.

Text Correction (7 of 9)

There was an error in the original publication [1]. The wrong in-text citations were referenced.

A correction has been made to Discussion, Prevalence of Deficiency of Other Essential Trace Elements *(*4.5.5. Manganese), Paragraph 1:

Our samples had a similar range of Mn in the whole blood of ewes (5.9–228 ng/mL) as the study by Ademi et al. [27] (6.0–243 ng/mL). When using the literature referenced by Ademi et al. [27] of 70–200 ng/mL, both studies resulted in a high (>96%) inadequacy rate. However, the whole blood reference range provided by Puls [25] of 20–40 ng/mL would indicate an inadequacy rate of 19% and an adequacy rate of at least 52.5% in our sheep samples. Symptoms of toxicity or deficiency of Mn are poorly characterized in mature sheep, and despite being an essential element, a wide range of Mn statuses appears to be well-tolerated [25,44]. For example, the threshold for toxicity in ppm of the diet is 30- to 80-fold higher than that of the adequate level [25]. Our results indicate that the reference range for whole blood Mn in sheep, as provided by Puls [25], is applicable. However, this range may warrant further expansion given the absence of Mn deficiency symptoms in the sheep we surveyed and the wide degree of tolerance described in the literature [25,44].

Text Correction (8 of 9)

There was an error in the original publication [1]. The wrong in-text citations were referenced.

A correction has been made to Discussion, Prevalence of Deficiency of Other Essential Trace Elements (4.5.6. Zinc), Paragraph 1:

According to the literature values, there was a high rate of Zn inadequacy in the sheep we surveyed, but it is unlikely the animals truly experienced nutritional Zn deficiency. The range of Zn detected in our study (0.94–4.72 µg/mL) was similar to the one reported by Ademi et al. [27] (1.4–3.8 µg/mL). Symptoms of Zn deficiency include anorexia, skeletal and reproductive disorders, impairment of the immune system, weak hoof walls, and poor skin and wool [25,44], which were not observed in the surveyed sheep. Owing to the fact that no Zn retention pool has been identified in any mammal, whole blood and other bodily tissues are not ideal matrices for diagnosing adequacy or deficiency [25,71]. Instead, Zn homeostasis appears to be controlled by changes in gastrointestinal absorption and excretion [72]. Serum and plasma are the preferred matrices for maximizing accuracy in diagnosing Zn status but are not without limitation; age, sex, time of day, recent dietary intake, stress, illness, and a plethora of other intrinsic factors can influence Zn levels [73]. Alternative tests have been proposed to improve accuracy in Zn status diagnosis, such as measuring hair Zn accumulation or measuring the abundance of linoleic acid relative to dihomo-γ-linoleic acid (DGLA), as the conversion of linoleic acid to DGLA is a Zn-dependent process [74]. Given these complexities, relying solely on whole blood Zn concentrations may lead to the misclassification of deficiency [75]. Until improved tests become available, maintaining adequate Zn in the diet of sheep and testing plasma or serum when Zn deficiency is suspected remains the accepted recommendation.

Text Correction (9 of 9)

There was an error in the original publication [1]. The wrong in-text citations were referenced.

A correction has been made to Discussion, Other Potentially Essential or Toxic Elements in Sheep Whole Blood, Paragraph 1:

The nutritive value of Al, As, B, Ba, Be, Cd, Cr, Hg, Pb, Tl, and V remains unestablished in sheep and several of these minerals are associated with toxicity [25,38,44,76–79]. All of these minerals exist at low levels in U.S. soil under normal circumstances [25,80]. For Al, B, Ba, Be, Cr, Tl, and V, a whole blood threshold in sheep has not been established, which poses a challenge for interpreting the clinical relevance of our results; however, none of the sheep were suspected of any mineral toxicity. While maximum tolerable levels in the diet of ruminants have been described in the literature, an association between toxic dietary levels and whole blood accumulation has not been made [25,44]. It would be highly beneficial to establish tolerable levels of potentially essential or toxic elements in the whole blood of sheep. However, careful consideration must be given to the specific form and metabolized state of each element, as not all forms pose the same risk of toxicity (e.g., Al, Ba, Hg) [25,81].

The authors state that the scientific conclusions are unaffected.

This correction was approved by the Academic Editor. The original publication has also been updated.
