# Supplementary material for: Status of Selenium and Other Essential and Toxic Elements in Oregon Grazing Sheep
Source: Animals (Basel). 2025 Jun 18;15(12):1799. doi: 10.3390/ani15121799 (PMC12190036; doi:10.3390/ani15121799)
Supplement: Supplementary file 1 [file animals-15-01799-s001.zip › Suppl File 2 Questionnaire.pdf]

Oregon State University
College of Agricultural Sciences

**Daniella Hasan**
Bionaz Lab

# Sheep Producer Micromineral Preassessment

Full Name: ______________________________
Address: ______________________________
______________________________
Phone: ______________________________
Email: ______________________________
Date: ______________________________

Describe the purpose(s) of your operation (wool, meat, hobby, grass fed, etc.):

______________________________
______________________________

**Diet** (please specify feed type and brand if applicable)
*Example: alfalfa pellets, local pasture, Purina all-stock*
*Seasonal: Rolled oats Dec-Jan*

Lambs: ______________________________
Weanlings: ______________________________
Seasonal: ______________________________
Dry ewes: ______________________________
Seasonal: ______________________________
Pregnant ewes:
Early: ______________________________
Mid: ______________________________
Late: ______________________________
Lactating ewes: ______________________________

Comments or further specification:

______________________________
______________________________
______________________________
______________________________
______________________________
______________________________

Oregon State University
College of Agricultural Sciences

**Daniella Hasan**
Bionaz Lab

**Minerals**

Do you use BO-SE?

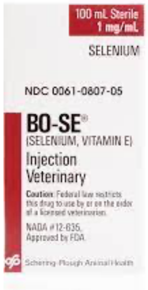


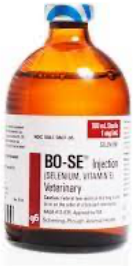


If so, when:

How much:

Do you use mineral blocks or loose mineral or protein tubs?

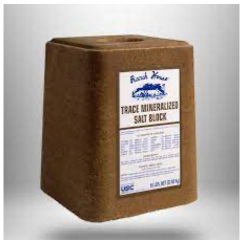


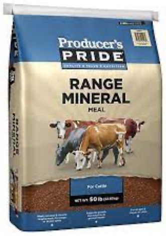


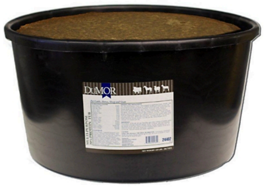


How often do animals have access to it:

Type/Brand:

Do you use selenium fertilization on pastures? **Yes** ☐ **No** ☐

Describe any health or production concerns you have:

______________________________________________________________
______________________________________________________________
______________________________________________________________
______________________________________________________________

Please use this scale to answer the following:

| **1** Extremely Deficient | **2** Moderately Deficient | **3** Mildly Deficient | **4** Slightly Deficient | **5** Sufficient | **6** Slightly too much | **7** Mildly too much | **8** Moderately too much | **9** Extremely too much |
|---|---|---|---|---|---|---|---|---|

Where do you believe your sheep are in terms of selenium status? _________
Where do you believe your sheep are in terms of copper status? _________
Are you concerned about any mineral toxicities or heavy metal accumulation? **Yes** ☐ **No** ☐
If so, which? ______________________________________________________________

Oregon State University
College of Agricultural Sciences

**Daniella Hasan**
Bionaz Lab
[redacted]

May I contact you for further information? **Yes** ☐ **No** ☐

May I contact you with test results? **Yes** ☐ **No** ☐

Please, select minimum of 3 and maximum of 10 female sheep with at the least 1 lambing.

| **Animal ID** | **Breed** | **Age** | **Date last lambing** | **Body Condition Score (1-5)** | **Pregnant (Yes/No)** | **Lactating (Yes/No)** |
|---|---|---|---|---|---|---|
| | | | | | | |
| | | | | | | |
| | | | | | | |
| | | | | | | |
| | | | | | | |
| | | | | | | |
| | | | | | | |
| | | | | | | |
| | | | | | | |
| | | | | | | |

Breed: if a crossbred indicate all breeds
