# Supplementary material for: Status of Selenium and Other Essential and Toxic Elements in Oregon Grazing Sheep
Source: Animals (Basel). 2025 Jun 18;15(12):1799. doi: 10.3390/ani15121799 (PMC12190036; doi:10.3390/ani15121799)
Supplement: Supplementary file 1 [file animals-15-01799-s001.zip › Supplementary Figures.pdf]

**Supplementary Figures**

***Supplementary Figure S1:*** Results for 21 analyses of NIST 1577b (blue bars) with 2$\sigma$ error bars compared to accepted values and uncertainties (orange bars) for 11 elements measured in this study. Values in dark boxes represent the difference in our measured values from the accepted (in percent). Values at the bottom in grey boxes represent the certified values in the NIST 1577b standard material.

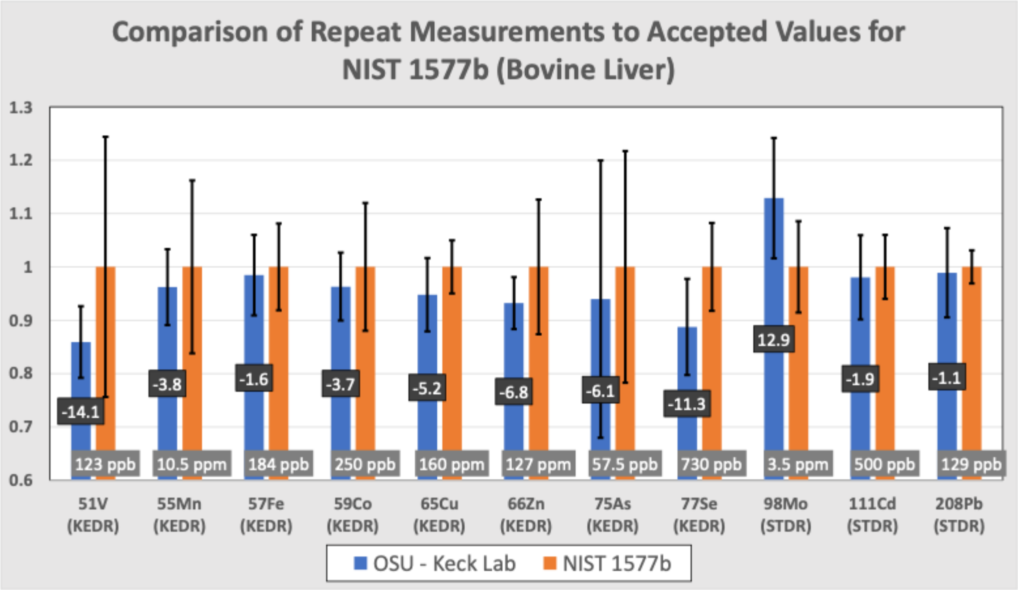

***Supplementary Figure S2:*** Status of Al, B, Ba, Be, and Cr in whole blood arranged by farm according to mean Se rank. Samples above the LOD for each element included. The dashed line indicates overall mean of each element by farm. Bars indicate the 10$^{th}$ and 90$^{th}$ percentiles.

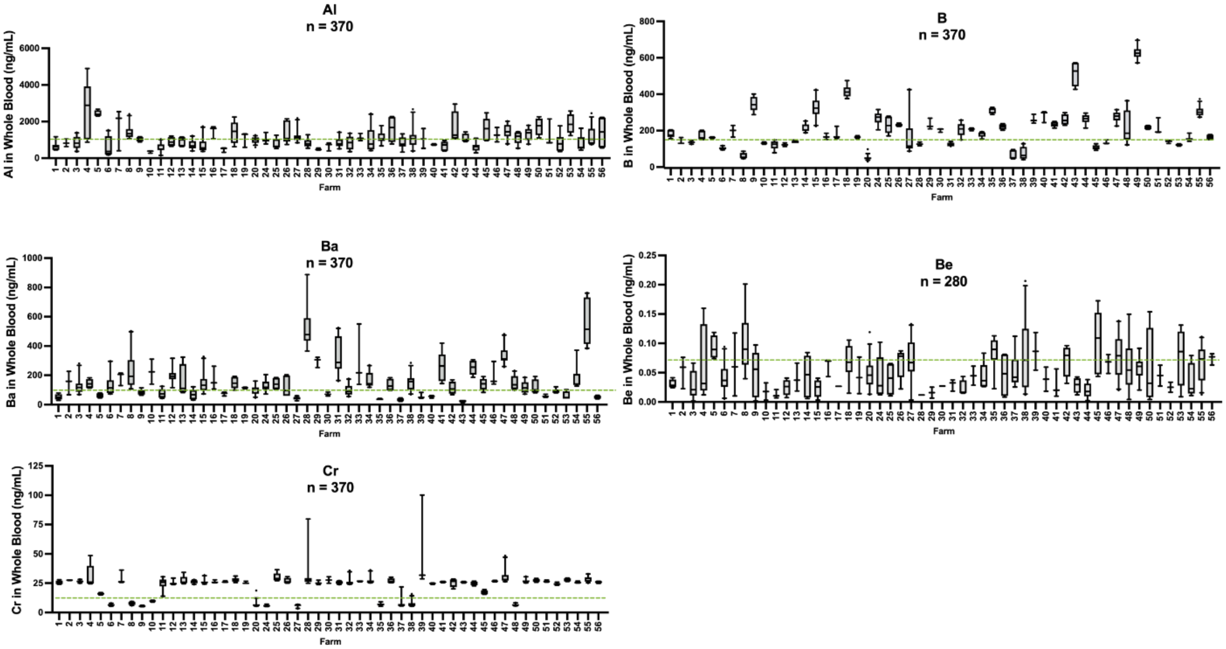

***Supplementary Figure S3:*** Farmers' (n = 42) response to perceived Se status (***Suppl. Figure 3a.***) on a nine-point scale (***Suppl. Figure 3b.***) answered prior to receiving result. Farms are ranked by mean flock whole blood Se (25.5-394 ng/mL).

**a.**

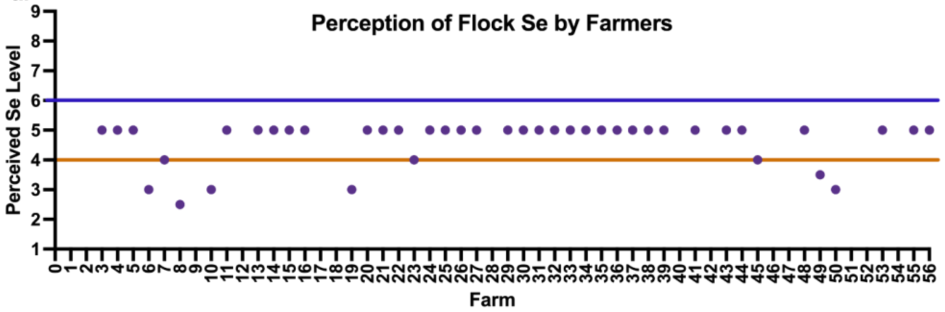


**b.**

Please use this scale to answer the following:

| 1 | 2 | 3 | 4 | 5 | 6 | 7 | 8 | 9 |
|---|---|---|---|---|---|---|---|---|
| Extremely Deficient | Moderately Deficient | Mildly Deficient | Slightly Deficient | Sufficient | Slightly too much | Mildly too much | Moderately too much | Extremely too much |
